# Remote Antimicrobial Stewardship in Community Hospitals

**DOI:** 10.3390/antibiotics4040605

**Published:** 2015-11-13

**Authors:** Zachary H. Wood, Nicole C. Nicolsen, Nichole Allen, Paul P. Cook

**Affiliations:** 1 Brody School of Medicine, 600 Moye Blvd, Greenville, NC 27834, USA; E-Mail: cookp@ecu.edu; 2 Vidant Medical Center, 2100 Stantonsburg Rd, Greenville, NC 27834, USA; E-Mails: Nicole.Nicolsen@vidanthealth.com (N.C.N.); nallen@vidanthealth.com (N.A.)

**Keywords:** antibiotics, antimicrobials, electronic medical record, stewardship, community hospitals

## Abstract

Antimicrobial stewardship has become standard practice at university medical centers, but the practice is more difficult to implement in remote community hospitals that lack infectious diseases trained practitioners. Starting in 2011, six community hospitals within the Vidant Health system began an antimicrobial stewardship program utilizing pharmacists who reviewed charts remotely from Vidant Medical Center. Pharmacists made recommendations within the electronic medical record (EMR) to streamline, discontinue, or switch antimicrobial agents. Totals of charts reviewed, recommendations made, recommendations accepted, and categories of intervention were recorded. Linear regression was utilized to measure changes in antimicrobial use over time. For the four larger hospitals, recommendations for changes were made in an average of 45 charts per month per hospital and physician acceptance of the pharmacists’ recommendations varied between 83% and 88%. There was no significant decrease in total antimicrobial use, but much of the use was outside of the stewardship program’s review. Quinolone use decreased by more than 50% in two of the four larger hospitals. Remote antimicrobial stewardship utilizing an EMR is feasible in community hospitals and is generally received favorably by physicians. As more community hospitals adopt EMRs, there is an opportunity to expand antimicrobial stewardship beyond the academic medical center.

## 1. Introduction

The overuse and misuse of antimicrobial agents is a global problem that has led to the development of antimicrobial resistance in both the hospital and community setting. One of the primary strategies for combating resistance is through the use of antimicrobial stewardship programs (ASPs) [1,2]. The ASP’s primary goal includes optimizing antimicrobial use through reduction of unnecessary antimicrobial use and confirmation of proper antimicrobial use (including drug, dose, route, and duration), in order to achieve the best clinical outcomes. While ASPs have been proven effective in both academic hospitals [3] and smaller community hospitals [4], small community hospitals tend to have more difficulty establishing these programs. These difficulties include staffing constraints, lack of funding, and lack of administrative and medical staff support [5].

Vidant Medical Center (VMC) has had a successful ASP in place since 2001. The ASP uses a primary strategy of prospective audit with feedback. Given the success of the ASP at the tertiary care center, we expanded the ASP to six of the seven community hospitals within the Vidant Health (VH) system. In December 2011 the ASP was implemented at Vidant Roanoke-Chowan Hospital (VROA), in March 2012 at Vidant Bertie Hospital (VBER), Vidant Chowan Hospital (VCHO), and The Outer Banks Hospital (OBH), in October 2013 at Vidant Duplin Hospital (VDUP), and in December 2013 at Vidant Beaufort Hospital (VBEA). We were able to accomplish this process through use of the electronic medical record (EMR) Epic (Madison, WI, USA), which is shared across the VH system [6]. To date, we are unable to locate any previous attempt at managing an ASP via EMR and central monitoring. We currently collect data on intervention outcomes, cost savings, physician acceptance rates, number of charts reviewed, number of recommendations made, anti-methicillin-resistant *Staphylococcus aureus* (MRSA) drug use, anti-pseudomonal drug use, broad spectrum drug use, and total antimicrobial drug use.

## 2. Methods

### 2.1. Central ASP at VMC

VMC is a 909-bed, tertiary-care academic medical center affiliated with the Brody School of Medicine at East Carolina University (Greenville, NC, USA). VMC’s ASP was established in 2001 and has been reviewed in previous publications [7,8]. This ASP was formed by the Antimicrobial Utilization & Stewardship Subcommittee (AUSS) and was approved by the Pharmacy and Therapeutics Committee and the medical staff executive committee. When the ASP first started it had an infectious diseases physician director and one pharmacist (1 full time equivalent [FTE]), with an additional pharmacist added in 2004 (0.5 FTE) and two additional pharmacists added when the program expanded to the community hospitals (1.5 FTE). All members of the ASP are physically located at VMC. The VH ASP fully operates five days per week with on-call and follow-up services provided on the weekend. Patient chart review for VMC is completed by one of the pharmacists for all adult patients that have been on a restricted or controlled antibiotic for ≥72 h.

### 2.2. Expansion of the ASP to Vidant Community Hospitals

VMC is the flagship hospital of the VH system. Including VMC, VH currently has eight hospital locations across eastern North Carolina with six of seven community hospitals being a part of this ASP. These six community hospitals are described in Table 1. None of the community hospitals within this ASP have infectious diseases consult services. While some of these hospitals do have a few order sets that were in place prior to the ASP, there are no formal infection related guidelines at any of these hospitals. To initiate implementation of an ASP at each community hospital, the physician director and pharmacist representative(s) visited each hospital to describe the program, discuss specifics, and begin to build relationships with the local physicians, pharmacists, infection control practitioners, and microbiology staff.

**Table 1:** Characteristics of Vidant community hospitals.

Hospital Name	Beds	ASP Start Date	Services
Vidant Beaufort Hospital, Washington, NC (Hospital A)	142	December 2013	medical, surgical, intensive care, emergency, and orthopedics
Vidant Chowan Hospital, Edenton, NC (Hospital B)	49	March 2012	medical, surgical, intensive care, emergency, and orthopedics
Vidant Duplin Hospital, Kenansville, NC (Hospital C)	81	October 2013	medical, surgical, intensive care, emergency, and orthopedics
Vidant Roanoke-Chowan Hospital, Ahoskie, NC (Hospital D)	114	December 2011	medical, surgical, intensive care, emergency, orthopedics, and wound care
Vidant Bertie Hospital, Windsor, NC (Hospital E)	6	March 2012	medical and emergency
The Outer Banks Hospital, Nags Head, NC (Hospital F)	21	March 2012	medical, surgical, emergency, and orthopedics

At these hospitals, any adult patient that receives a controlled antimicrobial for ≥24 h triggers a chart review by the ASP pharmacist and is listed on the EMR-generated report that is run daily Monday through Friday. A full list of all controlled antimicrobials can be found in Table 2. The formulary restriction program in place at VMC is not currently in place at the community hospitals. The time window for antibiotic use that triggered a chart review was shortened from 72 h to 24 h at the community hospitals after it was noticed that the length of stay at the community hospitals was generally shorter than it is at VMC. Based on microbiology culture results, radiology reports, and the working diagnosis, the pharmacist, with input from the physician director, makes recommendations to change or stop the controlled antimicrobial agent(s) by leaving a note in the EMR. These recommendations are generally based off of the guidelines published by the Infectious Diseases Society of America (IDSA). After a note is left, the physician can make the recommended change(s) on his/her own or reply in a progress note or as an addendum to the ASP note with the reason why current therapy will continue. After 24 h, if the recommendation is not acknowledged by the primary provider then the ASP pharmacist implements the recommendation per protocol as a telephone order from the ASP physician director.

**Table 2:** Antimicrobials classified as controlled by the antimicrobial stewardship program for community hospitals.

Controlled Antimicrobials	
Acyclovir	Fidaxomicin
Amikacin	Fluconazole
Amphotericin B lipid complex	Flucytosine
Ampicillin/sulbactam	Ganciclovir
Azithromycin	Linezolid
Aztreonam	Meropenem
Cefepime	Micafungin
Cefotaxime	Moxifloxacin
Ceftaroline	Piperacillin/tazobactam
Ceftriaxone	Posaconazole
Ciprofloxacin	Tedizolid
Clindamycin	Tigecycline
Colistimethate (or colistin)	Tobramycin
Dalbavancin	Vancomycin
Daptomycin	Voriconazole
Ertapenem	Non-formulary antibiotics

### 2.3. ASP Data Collection

Antimicrobial drug use was measured for each hospital for all antimicrobials used in defined daily dose per 1000 patient-days (DDD/1000 PD) according to World Health Organization (WHO) standards (http://www.whocc.no/atcddd/). This drug usage included anti-fungals, anti-virals, and anti-bacterial agents, including drugs that are not considered controlled and that are not evaluated by the ASP. Certain antimicrobials are also divided into additional categories. Anti-pseudomonal agents included ceftazidime (not on formulary), cefepime, piperacillin/tazobactam, meropenem, doripenem (not on formulary), imipenem (not on formulary), ciprofloxacin, levofloxacin (not on formulary), aminoglycosides, and aztreonam. Anti-MRSA agents included ceftaroline, clindamycin, daptomycin, dalbavancin, doxycycline, linezolid, tedizolid, tigecycline, trimethoprim/sulfamethoxazole, and vancomycin. This data was collected by the ASP on a quarterly basis and was used to trend usage over time. 

Each recommendation left by the ASP and accepted by the primary provider was then classified under one of nine different types of intervention. The intervention types included: additional test required to make diagnosis, adverse event avoided, antibiotic-pathogen matched, dose adjusted, empiric antibiotic recommendation, drug discontinued, intravenous (IV) to oral (PO) switch, de-escalation of therapy, and indwelling urinary catheter discontinued or changed. Each quarter we determined how many of the accepted interventions fell into each of these categories. For all of the interventions that resulted in a drug being discontinued, we estimated a cost-avoidance for the hospital determined by the institutional acquisition cost of the drug and the number of days of therapy spared (assuming the initial order continued through completion of a seven day course). Seven days was used as the default duration because all antimicrobial orders are automatically given a duration of seven days in the EMR. Patients who had drugs discontinued but were then discharged are documented separately. The number of charts reviewed, number of recommendations made (percent intervention), and number of recommendations accepted by the primary provider (physician acceptance rate) were also collected for each hospital on a monthly basis.

### 2.4. Surveillance Definitions

Nosocomial Gram-negative and Gram-positive data sets were created by querying MedMined^®^ (CareFusion, Birmingham, AL, USA). These definitions are the same as those currently used at VMC [6]. All clinical care unit specimens (blood, sterile fluid, sputum, urine, wounds and anaerobic specimens) taken between 1 July 2010 and 30 June 2015 from hospitals A–F were included. Percent susceptible was defined as the percentage of total isolates that were susceptible to the selected antimicrobial. Intermediately susceptible isolates were classified as resistant. Susceptibility profiles were compared on a year-by-year basis.

### 2.5. Epic

Currently every hospital in the VH system uses Epic. Each hospital’s remote ASP uses the same process and reporting as VMC [6]. First, an electronic progress note with the ASP recommendation was entered into the EMR. The ASP pharmacist then entered a unique order into the system entitled “antimicrobial management”. This order functioned as a best practice alert. Whenever a physician or other provider logged into a patient’s chart, the EMR automatically opened a new window with a message from the ASP to the provider. This communication window alerted the provider that the ASP had left a recommendation in the EMR. The provider then had 24 h to respond to (*i.e.*, accept or reject) the recommendation per medical staff guidelines. At this time, the “antimicrobial management” order was discontinued. Internally written reports from the Epic reporting manual were used to identify patients and collect usage, outcome, and workload data.

### 2.6. Statistics

Statistical analysis was performed using IBM SPSS Statistics versions 22 and 23 (IBM Corp., Armonk, NY, USA). Linear regression was used to examine antimicrobial use and antimicrobial susceptibility from ASP implementation date through June 2015. A *p*-value of ≤0.05 was considered significant.

## 3. Results

### 3.1. Workload and Physician Acceptance

Total number of charts reviewed, recommendations made, and recommendations accepted are in Table 3. The first month for each hospital was omitted due to the beginning of the ASP being in the middle of the month. The average number of charts reviewed per month ranged from 17 to 148. The percent of recommendations made per charts reviewed per month ranged from 40% to 63%. The percent of recommendations accepted per month by the physician ranged from 81% to 95%.

**Table 3:** Monthly antimicrobial stewardship program (ASP) activity (first full month of ASP through June 2015).

Hospital	A	B	C	D	E	F
First full month of ASP	January 2014	April 2012	November 2013	January 2012	April 2012	April 2012
Adult inpatient days	14,840	17,134	11,379	41,169	5017	9531
Total number of charts reviewed	1563	1753	1179	6797	669	943
Average number of charts reviewed/month	87	45	59	148	17	24
Average number of recommendations/month	39	25	24	93	9	12
Average number of recommendations accepted/month	33	20	21	78	8	9
Recommendations/charts reviewed (%) *	45%	57%	40%	63%	54%	47%
Recommendations accepted (%) *	83%	85%	88%	87%	95%	81%

* Based on actual numbers not averages.

### 3.2. Intervention Outcomes and Cost Savings

Classification of outcomes for accepted interventions between January 2014 and June 2015 are in Table 4. The recommendations by the ASP most often resulted in antimicrobial drug discontinuation. Hospital drug cost savings were also calculated for each drug discontinued using the days of antibiotic therapy avoided and multiplying by the institutional acquisition cost of antibiotic therapy per day. As mentioned above, seven days was used as the default assumed duration because all antimicrobial orders are automatically given a duration of seven days in the EMR. The total cost savings associated with the drugs discontinued between January 2014 and June 2015 were as follows: hospital A, $16,928; hospital B, $7008; hospital C, $5887; hospital D, $53,618; hospital E, $4309; hospital F, $1616. These cost savings reflect only drug cost and do not account for other costs savings which include but are not limited to IV supplies, nursing time, pharmacy time, or avoidance of opportunistic infections. Patients who were discharged without antimicrobial therapy are documented separately, since there is no cost avoidance for the hospital. The number of patients who were discharged without antimicrobial therapy between January 2014 and June 2015 were as follows: hospital A, 57 patients; hospital B, 35 patients; hospital C, 30 patients; hospital D, 154 patients; hospital E, 14 patients; hospital F, 14 patients.

**Table 4:** Accepted interventions from January 2014 through June 2015 by hospital.

Intervention (Number over Past 18 Months)	A	B	C	D	E	F
Additional Test Required to Make Diagnosis	9	2	2	24	4	2
Adverse Event Avoided	34	10	21	45	7	9
Antibiotic-Pathogen Matched	31	20	27	68	5	3
Dose Adjusted	76	33	51	106	7	10
Empiric Antibiotic Recommendations	50	48	40	128	15	11
Drug Discontinued	279	168	132	747	78	50
IV to PO	100	84	63	345	43	32
Foley Discontinued or Changed	2	1	2	2	1	0
De-escalation of therapy	38	17	23	105	7	12

**Table 5:** Changes in the use of various categories of antimicrobial agents for hospitals A–F measured in DDD/1000 PD.

Class	Jan–Mar 2011	Apr–Jun 2011	Jul–Sept 2011	Oct–Dec 2011	Jan–Mar 2012	Apr–Jun 2012	Jul–Sept 2012	Oct–Dec 2012	Jan–Mar 2013	Apr–Jun 2013	Jul–Sept 2013	Oct–Dec 2013	Jan–Mar 2014	Apr–Jun 2014	Jul–Sept 2014	Oct–Dec 2014	Jan–Mar 2015	Apr–Jun 2015	*p*-Value
**Hospital A**												↓							
Quinolones	-	-	-	-	-	-	-	-	176	157	156	161	155	157	112	178	159	128	N/S
Cephalosporins	-	-	-	-	-	-	-	-	205	188	206	204	185	209	238	250	237	291	* 0.003
Macrolides	-	-	-	-	-	-	-	-	125	229	194	150	270	100	115	124	99	143	N/S
Anti-Pseudomonal	-	-	-	-	-	-	-	-	248	231	267	210	228	239	259	254	201	226	N/S
Anti-MRSA	-	-	-	-	-	-	-	-	265	271	249	244	276	342	247	301	233	334	N/S
Total	-	-	-	-	-	-	-	-	1043	1199	1230	1141	1275	1329	1219	1243	1108	1245	N/S
**Hospital B**					↓														
Quinolones	-	-	378	420	370	354	250	220	114	110	108	161	155	156	141	195	177	161	* 0.001
Cephalosporins	-	-	338	410	387	360	384	333	322	372	319	291	399	355	348	374	334	428	N/S
Macrolides	-	-	226	295	265	310	226	275	334	228	236	228	271	208	186	334	301	297	N/S
Anti-Pseudomonal	-	-	630	554	470	513	369	361	170	184	166	175	226	199	236	265	205	309	* 0.001
Anti-MRSA	-	-	294	267	224	361	276	221	229	307	246	264	296	338	322	252	256	324	N/S
Total	-	-	1750	1772	1623	1918	1492	1378	1334	1405	1259	1281	1526	1465	1436	1640	1513	1671	N/S
**Hospital C**												↓							
Quinolones	-	-	-	-	-	-	-	137	131	141	122	182	147	129	133	150	141	128	N/S
Cephalosporins	-	-	-	-	-	-	-	330	318	275	270	323	305	372	426	342	294	331	N/S
Macrolides	-	-	-	-	-	-	-	372	324	305	273	259	280	227	205	259	206	211	* <0.001
Anti-Pseudomonal	-	-	-	-	-	-	-	174	134	213	176	161	175	167	183	183	202	200	N/S
Anti-MRSA	-	-	-	-	-	-	-	306	434	371	366	511	336	399	389	360	282	258	N/S
Total	-	-	-	-	-	-	-	1438	1448	1476	1352	1573	1484	1507	1498	1494	1292	1352	N/S
**Hospital D**				↓															
Quinolones	296	262	287	222	230	188	190	169	138	122	133	179	154	135	119	119	132	101	* <0.001
Cephalosporins	235	183	217	226	209	291	197	194	214	241	247	290	252	316	330	280	331	393	* <0.001
Macrolides	163	100	102	120	159	102	106	135	135	113	104	144	199	140	143	166	171	163	* 0.029
Anti-Pseudomonal	482	470	448	385	317	311	280	280	208	217	212	233	211	256	226	215	244	280	* <0.001
Anti-MRSA	275	280	306	269	213	300	264	244	235	263	262	375	254	378	271	249	290	252	N/S
Total	1282	1164	1221	1136	1045	1214	1090	1093	1005	1038	1062	1373	1167	1355	1195	1277	1349	1353	N/S
**Hospital E**					↓														
Quinolones	-	624	580	537	584	406	372	250	226	217	168	152	281	237	195	218	251	204	* <0.001
Cephalosporins	-	428	315	410	495	412	443	435	128	467	480	362	391	427	492	681	581	758	* 0.021
Macrolides	-	286	210	393	355	312	215	577	744	302	264	299	492	640	478	790	632	575	* 0.008
Anti-Pseudomonal	-	697	674	609	656	483	428	409	82	220	303	181	199	276	197	255	143	388	* <0.001
Anti-MRSA	-	119	268	256	236	416	243	286	385	257	269	357	231	337	296	339	352	279	N/S
Total	-	1810	1627	1870	2169	1723	1542	1978	1653	1607	1486	1647	1788	2041	1810	2426	2235	2491	N/S
**Hospital F**					↓														
Quinolones	-	-	-	-	328	419	#	350	329	362	359	367	356	379	424	262	270	327	N/S
Cephalosporins	-	-	-	-	518	379	#	760	460	520	523	516	494	619	572	533	423	487	N/S
Macrolides	-	-	-	-	215	179	#	394	283	297	430	233	241	362	322	182	260	270	N/S
Anti-Pseudomonal	-	-	-	-	305	431	#	446	242	316	418	273	325	423	516	352	344	402	N/S
Anti-MRSA	-	-	-	-	288	347	#	383	332	394	417	399	481	730	784	618	178	547	N/S
Total	-	-	-	-	1764	2006	#	2161	1695	2130	2456	1975	2153	2934	2933	2171	1783	2253	N/S

DDD/1000 PD, defined daily dose per 1000 patient-days; N/S, not significant; MRSA, methicillin-resistant *Staphylococcus aureus*; * Linear Regression: (*p* ≤ 0.05); Arrows indicate ASP start date for individual hospitals; # We were unable to calculate usage data for this quarter.

### 3.3. Antimicrobial Use

No hospital had a statistically significant change in total antimicrobial usage between their ASP start date and June 2015 (Table 5). Quinolone use decreased 57.4% in hospital B (*p* = 0.001), 65.9% in hospital D (*p* < 0.001), and 67.3% in hospital E (*p* < 0.001). Hospitals B, D, and E also had statistically significant decreases in anti-pseudomonal prescribing. No hospital had a statistically significant change in anti-MRSA prescribing rates. There were significant increases in cephalosporin use in hospitals A, D and E. Macrolide use decreased in hospitals C and D and increased in hospital E.

### 3.4. Antibiotic Susceptibility Profile

Only hospital D had enough isolates for statistical analysis. *P. aeruginosa* susceptibility was examined because this organism is a common nosocomial pathogen. Between 2011 and 2015, susceptibility of *P. aeruginosa* improved to ciprofloxacin (38% to 76%, *p* = 0.13), piperacillin-tazobactam (66% to 100%, *p* = 0.05), and meropenem (60% to 95%, *p* = 0.06) at hospital D (Table 6). In the same time period, *E. coli* susceptibility to ciprofloxacin improved from 38% in 2011 to 54% by 2015 (*p* = 0.19) (Table 6). There were no significant changes in rates of MRSA or *Clostridium difficile* infections at any of the facilities over the study period (data not shown).

**Table 6:** Susceptibility rates for selected antimicrobials and organisms at hospital D by year.

Antimicrobial	2011	2012	2013	2014	2015	*p*-Value
*E. coli*						
Ciprofloxacin	16/51 (38%)	28/56 (50%)	19/55 (34%)	26/44 (59%)	23/42 (54%)	0.19
*P. aeruginosa*						
Ciprofloxacin	12/31 (38%)	8/14 (57%)	23/28 (82%)	20/30 (66%)	20/26 (76%)	0.13
Piperacillin/tazobactam	20/30 (66%)	21/24 (84%)	20/27 (74%)	27/30 (90%)	23/23 (100%)	0.05
Meropenem	18/30 (60%)	21/24 (84%)	26/28 (92%)	28/30 (93%)	20/21 (95%)	0.06

The numerator represents the number of organisms that were susceptible to the given antibiotic and the denominator is the total number of organisms tested. The number in parentheses is the percentage of the total number that were susceptible to the given antibiotic.

## 4. Discussion

In 2007, the IDSA and the Society for Healthcare Epidemiology of America (SHEA) released guidelines for developing institutional programs to better antimicrobial stewardship through use of the EMR [9]. VH has demonstrated long-term beneficial effects of an ASP and has used the EMR as a means of optimizing antimicrobial use [6,8]. This study is unique in that we can find no record of any hospital system using their EMR to remotely practice antimicrobial stewardship at community hospitals.

Following expansion of the ASP to the community hospitals, 40%–63% of charts reviewed resulted in a recommendation being made with an 81%–95% physician acceptance rate. None of these recommendations occurred before the ASP was extended remotely to the community hospitals. This method provides an option for antimicrobial stewardship for smaller community hospitals and shows that physicians are willing to accept ASPs remotely.

The most commonly accepted intervention noted is drug discontinuation. This was associated with an average antimicrobial drug cost savings of $20,860.25 per hospital for the 4 largest hospitals over an 18 month period from January 2014 through June 2015. Again, this does not take into account additional cost savings that occur when adverse events are avoided, drugs are changed IV to PO, patient outcomes are optimized, and antimicrobial resistance is avoided.

One of the main targets with each remote ASP was the reduction of quinolone use due to its increased risk for both *Clostridium difficile* infections (CDI) and MRSA infections [10,11]. Overall, we saw statistically significant decreases in quinolone use at hospitals B, D and E but did not see significant changes at hospitals A, C or F. This decrease in quinolone use may be a driving factor for the decrease in anti-pseudomonal drug usage as well. Two possible reasons why hospitals A and C did not experience decreases in quinolone use could be due to the fact that their quinolone use was considerably lower at the beginning of ASP implementation and because the ASP is newer at both of these hospitals. 

Some hospitals did see an increase in cephalosporin use. This may be a result of implementation of a dose optimization protocol that attempted to maximize pharmacokinetic and pharmacodynamic properties for certain pathogens and patient populations. For example, all surgical cefazolin dosing was increased from 1 g to 2 g, empiric cefepime dosing for hospital acquired infections was increased from 1 g to 2 g every 12 h to 2 g every 8 h given by extended infusion, and ceftriaxone dosing was increased from 1 g to 2 g based on type of infection and patient specific parameters. While macrolide usage varied based on hospital, periodic analysis showed that most use of azithromycin is driven by the emergency department in the form of empiric sexually transmitted disease treatment or first dose for those not admitted to the hospital.

One important note to make is related to total antimicrobial use at each hospital. Overall, there was not a statistically significant decrease in total antimicrobial use at any of the community hospitals. The ASP does not review patients in the emergency department, those who come to the hospital daily for infusions, or those who are on antibiotics for less than the 24 h period it takes to flag on the report. However, all of this antimicrobial usage is included within the total usage reported. In addition, total usage includes antimicrobials that are not on the controlled list and that would never flag for ASP review.

The goal of ASPs is not only to reduce unnecessary use of antimicrobials, but also to improve resistance profiles. Because isolate numbers at each hospital were small, only hospital D’s isolate pool was large enough to analyze. There were improvements in antibiotic susceptibilities of *P. aeruginosa* to ciprofloxacin, piperacillin/tazobactam, and carbapenems over the four year time period.

Establishing a remote ASP is not without challenges. There can be variation in local resources including diagnostics and formulary. While the VH formulary is now standardized, there is still variation in what drugs are stocked by each pharmacy and there are currently no formulary restrictions at the community hospitals. Determining how to identify patients can also be a challenge and may have to be modified over time. Distinguishing cases that need stewardship assistance *vs.* a formal infectious diseases consult can also be a challenge.

Being a successful remote ASP does not stop with patient chart review. Continuing to develop relationships with the local staff (physicians, pharmacists, microbiology staff, and infection control practitioners) at the community hospitals is critical to improving patient care, as we view this as a team effort towards antimicrobial stewardship. The ASP pharmacists attempt to visit each community hospital on a yearly basis in order to provide some face-to-face interaction, conduct educational opportunities desired by the pharmacy or physician staff, share results of the program, and gather feedback. This process also allows formal ASP introduction to any new or temporary staff. It is common for acceptance rates of new physicians to be low until they become comfortable with the advantages of the program. In addition to daily chart review, the ASP has been responsible for tasks including, but not limited to, helping manage antimicrobial shortages and formulary, creating order sets, answering questions for the local wound care centers, and distributing a guide book that is updated yearly and includes key information about managing infectious diseases.

There are several limitations to this study. First, this study is based on aggregate data; the impact of the duration of antimicrobial use for an individual patient cannot be determined. Second, this dataset cannot correct for seasonal variation. Each ASP was implemented at a different time, with the oldest program running for four years and the youngest running for only one year. Because of these limitations, there were currently not enough data points to properly analyze antimicrobial patterns over the course of a year. Third, because of the small hospital sizes and small number of bacterial isolates, there was limited data regarding improvements in hospital antibiograms.

## 5. Conclusions

Remote ASPs utilizing the EMR provide an excellent alternative to the creation of new ASPs at small community hospitals with limited resources. Our data show that antimicrobial recommendations can be made and accepted at community hospitals at high percentages. Our data also show that we can potentially alter prescribing habits, save money, and change susceptibility patterns at community hospitals remotely as well. Overall, we have demonstrated successful implementation of a remote ASP through use of the EMR at small community hospitals.

## References

[1] Centers for Disease Control and Prevention (CDC) (2013). Antibiotic Resistance Threats in the United States.

[2] Fishman N. (2012). Policy statement on antimicrobial stewardship by the Society for Healthcare Epidemiology of America (SHEA). The Infectious Diseases Society of America (IDSA), and the Pediatric Infectious Diseases Society (PIDS). Infect. Control.

[3] Antworth A., Collins C.D., Kunapuli A., Klein K., Carver P., Gandhi T., Washer L., Nagel J.L. (2013). Impact of an antimicrobial stewardship program comprehensive care bundle on management of Candidemia. Pharmacotherapy.

[4] Trivedi K.K., Kuper K. (2014). Hospital antimicrobial stewardship in the nonuniversity setting. Infect. Dis. Clin. N. Am..

[5] Trivedi K.K., Rosenberg J. (2013). The state of antimicrobial stewardship programs in California. Infect. Control Hosp. Epidemiol..

[6] Cook P.P., Gooch M. (2015). Long-term effects of an antimicrobial stewardship programme at a tertiary-care teaching hospital. Int. J. Antimicro. Agents.

[7] Cook P.P., Catrou P.G., Christie J.D., Young P.D., Polk R.E. (2004). Reduction in broad-spectrum antimicrobial use associated with no improvement in hospital antibiogram. J. Antimicrob. Chemother..

[8] Cook P.P., Rizzo S., Gooch M., Jordan M., Fang X., Hudson S. (2011). Sustained reduction in antimicrobial use and decrease in methicillin-resistant *Staphylococcus aureus* and *Clostridium difficile* infections following implementation of an electronic medical record at a tertiary-care teaching hospital. J. Antimicrob. Chemother..

[9] Dellit T.H., Owens R.C., McGowan J.E., Gerding D.N., Weinstein R.A., Burke J.P., Huskins W.C., Paterson D.L., Fishman N.O., Carpenter C.F. (2007). Infectious Diseases Society of America and the Society for Healthcare Epidemiology of America guidelines for developing an institutional program to enhance antimicrobial stewardship. Clin. Infect. Dis..

[10] Dancer S.J. (2008). The effect of antibiotics on methicillin-resistant *Staphylococcus aureus*. J. Antimicrob. Chemother..

[11] Pepin J., Saheb N., Coulombe M.A., Alary M.E., Corriveau M.P., Authier S., Leblanc M., Rivard G., Bettez M., Primeau V. (2005). Emergence of fluoroquinolones as the predominant risk factor for *Clostridium difficile*-associated diarrhea: A cohort study during an epidemic in Quebec. Clin. Infect. Dis..

